# Integrating Periodontal Care and Orthodontic Treatment: A Case Study of a Patient Treated With Clear Aligner Therapy (CAT)

**DOI:** 10.1155/crid/1903921

**Published:** 2026-03-03

**Authors:** Waddah Sabouni, Jean-Philippe Mercier, Arthur Brincat

**Affiliations:** ^1^ Private Orthodontic Practice, Sanary-sur-Mer, France; ^2^ Private Dental Practice, Toulon, France

**Keywords:** clear aligner therapy, gingival recession, interdisciplinary orthodontics, invisalign, ortho-perio

## Abstract

Adult orthodontic treatment in patients with periodontal fragility requires careful interdisciplinary planning. This case report describes the interdisciplinary management of a 52‐year‐old female patient presenting with generalized gingival recessions (GRs), thin periodontal biotype, and dental crowding, treated using clear aligner therapy (CAT) combined with periodontal follow‐up and postorthodontic mucogingival surgery. Orthodontic treatment was carefully planned to reposition dental roots within the alveolar housing while minimizing periodontal risk in the absence of CBCT. Following orthodontic alignment and retention, connective tissue grafting was performed to treat residual GRs. Clinical, radiographic, and occlusal outcomes demonstrated stable periodontal conditions, changes in occlusal contact distribution, and satisfactory esthetic results at 1‐year follow‐up. The uniqueness of this case lies in the use of clear aligners as a periodontal‐supportive orthodontic strategy, combined with delayed surgical intervention, highlighting the role of precise root control and interdisciplinary coordination in periodontally fragile adult patients.

## 1. Introduction

The interaction between orthodontic tooth movement and periodontal health is particularly critical in adult patients presenting with gingival recession (GR) and reduced periodontal support. In such cases, orthodontic treatment requires careful planning to avoid exacerbating periodontal breakdown while aiming to improve tooth position and occlusal function [1–5].

Clear aligner therapy (CAT) has expanded its indications in recent years; however, precise root control remains challenging, especially in periodontally compromised patients. Previous studies have reported discrepancies between predicted and achieved movements, highlighting the need for adjunctive biomechanical strategies and careful staging when aligners are used in complex clinical situations [6–9].

The timing and sequencing of orthodontic and periodontal interventions play a key role in treatment outcomes. While periodontal surgery may be indicated before or after orthodontic treatment depending on the clinical situation, orthodontic tooth movement may contribute indirectly to periodontal tissue conditions by repositioning teeth within the alveolar envelope and creating more favorable conditions for subsequent periodontal healing [4, 5, 10].

While interdisciplinary approaches combining orthodontics and periodontal surgery have been reported, limited clinical data are available regarding the use of CAT as a preparatory and supportive step prior to mucogingival surgery in periodontally fragile adult patients. This case report aims to illustrate such an approach, highlighting the role of controlled orthodontic tooth movement, periodontal monitoring, and delayed surgical intervention in the management of GR, without the use of three‐dimensional imaging.

## 2. Clinical Case

A healthy, nonsmoking 52‐year‐old female patient with no relevant systemic diseases, no history of diabetes or autoimmune disorders, and no use of medications known to affect periodontal status sought orthodontic treatment using clear aligners to improve her smile.

**Figure 1:**
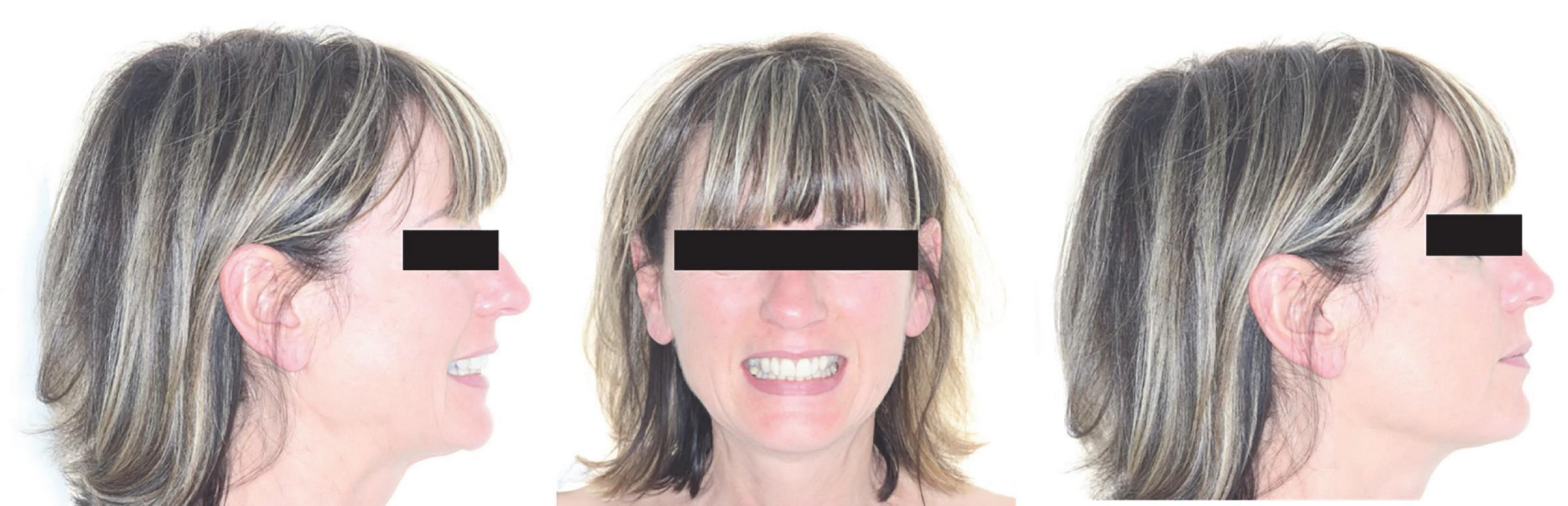
Pretreatment facial pictures.

**Figure 2:**
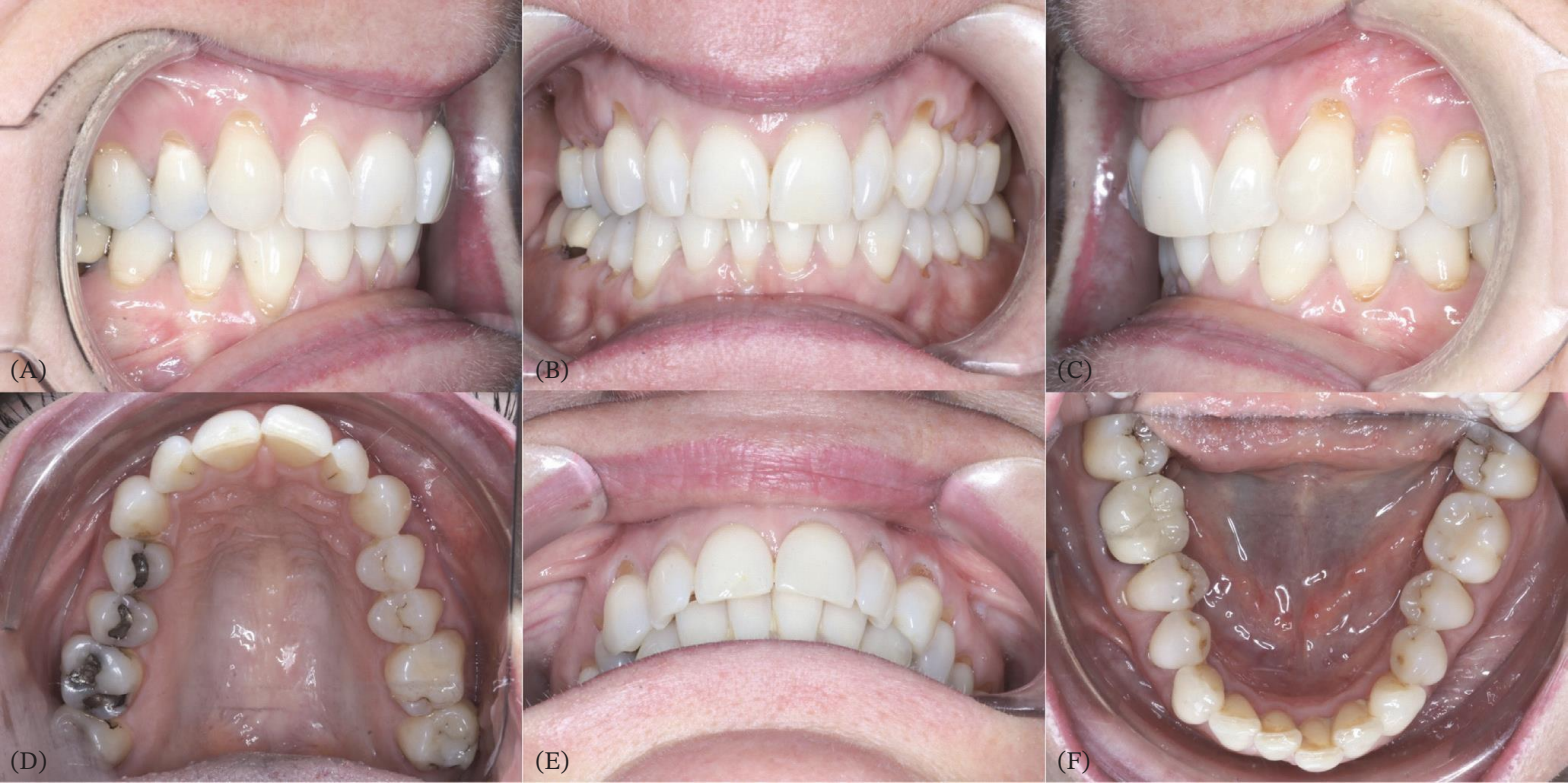
Pretreatment intraoral photo. Subfigures (A–C, E) show the patient’s occlusion before orthodontic treatment. Subfigures (D, F) show the arches.

Clinical evaluation uncovered multiple GRs on both dental arches, predominantly in the posterior upper jaw and the anterior lower jaw. These are classified as Class I lesions following Miller’s classification and RT2 lesions following Cairo’s classification [11]. The patient’s dental arches exhibited crowding (Figures 1 and 2).

Notably, at the enamel–cementum junction, particularly around the canines, there were signs of abfraction lesions (the B + type described by Pini‐Prato et al. [12]) indicating cervical tooth structure loss potentially due to mechanical stress from bruxism, which results in micro‐fractures of the enamel. Alternatively, these could be abrasion lesions caused by factors such as vigorous brushing [13].

The patient exhibited a thin gingival biotype with a reduced height of keratinized gingiva. Oral hygiene was generally satisfactory, except in areas where teeth were crowded, which fostered plaque accumulation and gingival inflammation.

**Figure 3:**
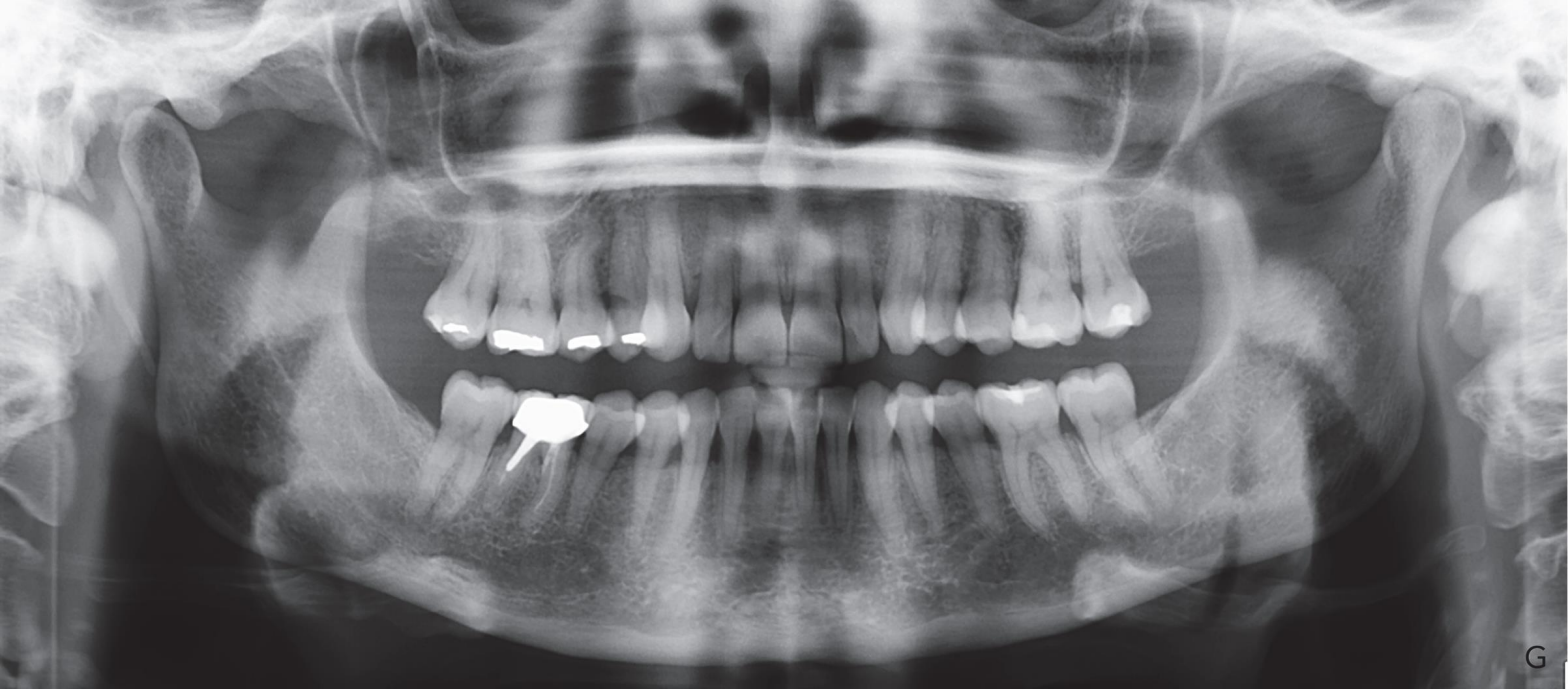
Pretreatment panoramic X‐ray.

Radiographic analysis (Figure 3) revealed slight generalized alveolar bone loss, the presence of a post‐and‐core crown on Tooth 46, and occlusal restorations mainly within Sector 1. Additionally, a mild asymmetry was observed in the right condyle. Given the moderate lower arch crowding, compressed arches, thin periodontal biotype, and slightly diminished bone support, it was concluded that periodontal surgical intervention would be necessary postorthodontic treatment with aligners. This approach aims to establish and maintain periodontal health in conjunction with the orthodontic realignment [14].

The objectives of this treatment plan included the alleviation of periodontal complications through comprehensive periodontal cleaning, the rectification of dental malocclusion to restore proper occlusion, the alleviation of arch compression in both the upper and lower jaws, and the restauration of the GR by surgical approach after orthodontic treatment [11, 13, 14].

The formulated treatment strategy comprised the steps shown in Table 1.

**Table 1:** Timetable of treatment strategy.

T1	Initial preparatory phase	Emphasis on enhancing the patient’s oral hygiene alongside nonsurgical periodontal therapy to establish a healthy baseline prior to orthodontic intervention
T2	Orthodontic phase	Implementation of orthodontic treatment utilizing aligners, continuously monitored by a periodontal specialist to ensure periodontal health is maintained. In that case, two sets of aligners were necessary to complete the treatment
T3	Retention phase	Implementation of retention strategies to preserve orthodontic outcomes
T4	Surgical intervention	Addressing gingival recessions surgically following orthodontic treatment
T5	Stability check‐up	The patient attends annual follow‐up visits; intraoral photos are made to assess the correct stability of the treatment and the retention devices

Based on these objectives, the predefined clinical outcomes of this case were the maintenance of periodontal stability throughout orthodontic treatment, the achievement of clinically acceptable root positioning within the alveolar envelope, the absence of periodontal deterioration, and the improvement of gingival conditions following subsequent mucogingival surgery.

This case underscores the imperative for a comprehensive, multidisciplinary approach in treating adult dental malocclusion, which necessitates close collaboration between orthodontists and periodontists to achieve enduring esthetic and functional results.

To maintain the positive outcomes and prevent recurrence, periodontal maintenance every 6 months was advised. Following occlusal restoration, alignment of the teeth within their bony support, and effective plaque control, a black triangle developed between Teeth 32 and 31 and 41. This was associated with increased GR around Teeth 31–41, without evidence of additional bone loss, corresponding to a Miller Class II or Cairo RT2 recession [11, 15]. There was an absence of keratinized gingiva on Tooth 41, with the labial frenum exerting traction on the marginal gingiva and opening the sulcus.

According to the objective of the patient, regenerative surgical intervention was deemed necessary postorthodontically to enhance tissue esthetics and support.

The aligner treatment was executed in two distinct phases. Treatment planning set‐up was made with the Clincheck pro software using 3D crown and root control tools. It should be noted that no CBCT was available to treat this patient: The initial phase was designed with 24 aligners with the application at the aligner number 4 optimized attachments for a better control of the movements, two power ridges on 21 and 42 helping the torque expression and bite ramps behind the four upper incisors was also added due to the overbite the patient presented.

**Figure 4:**
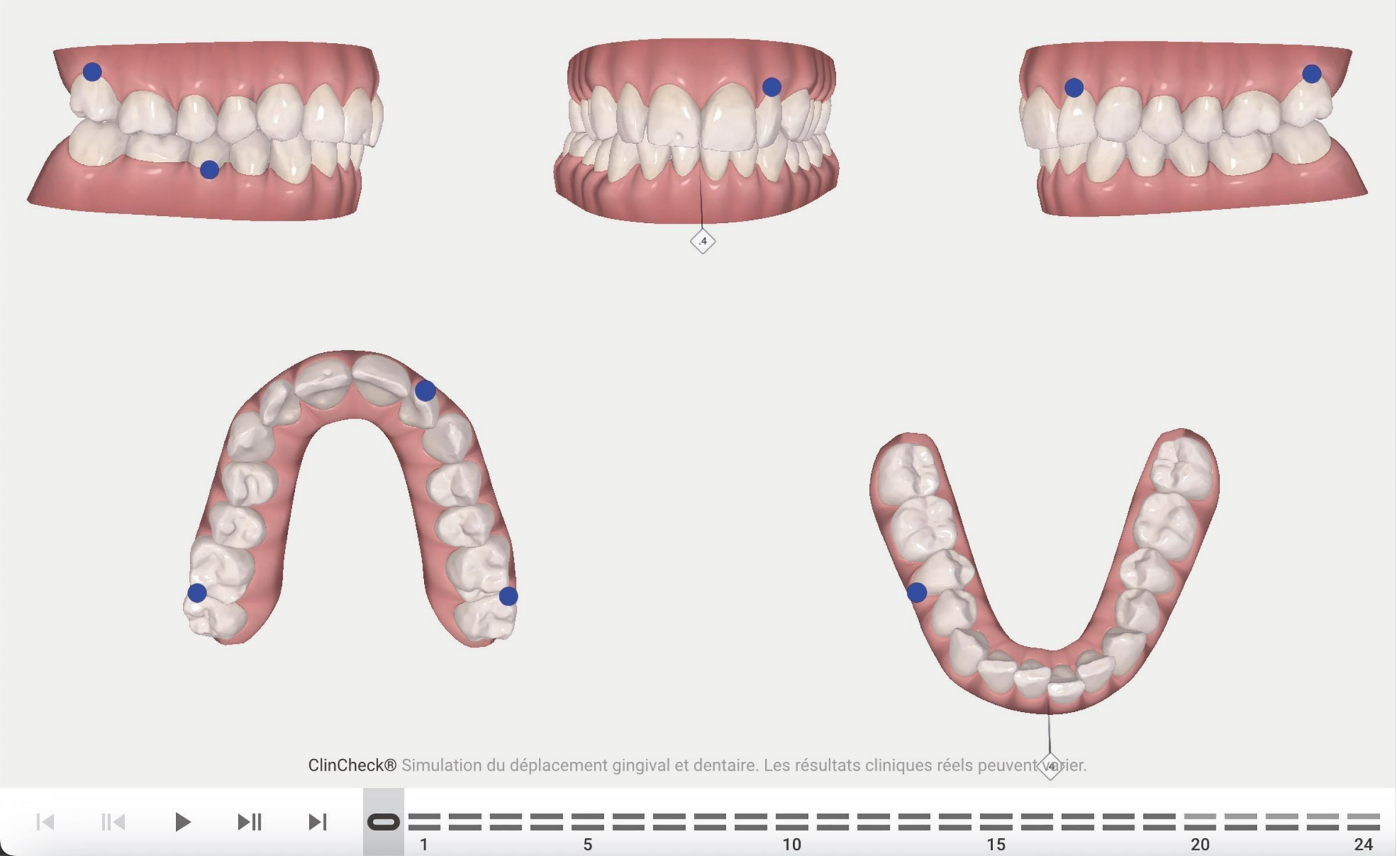
First Clincheck, patient’s occlusion at aligner *n*°0.

**Figure 5:**
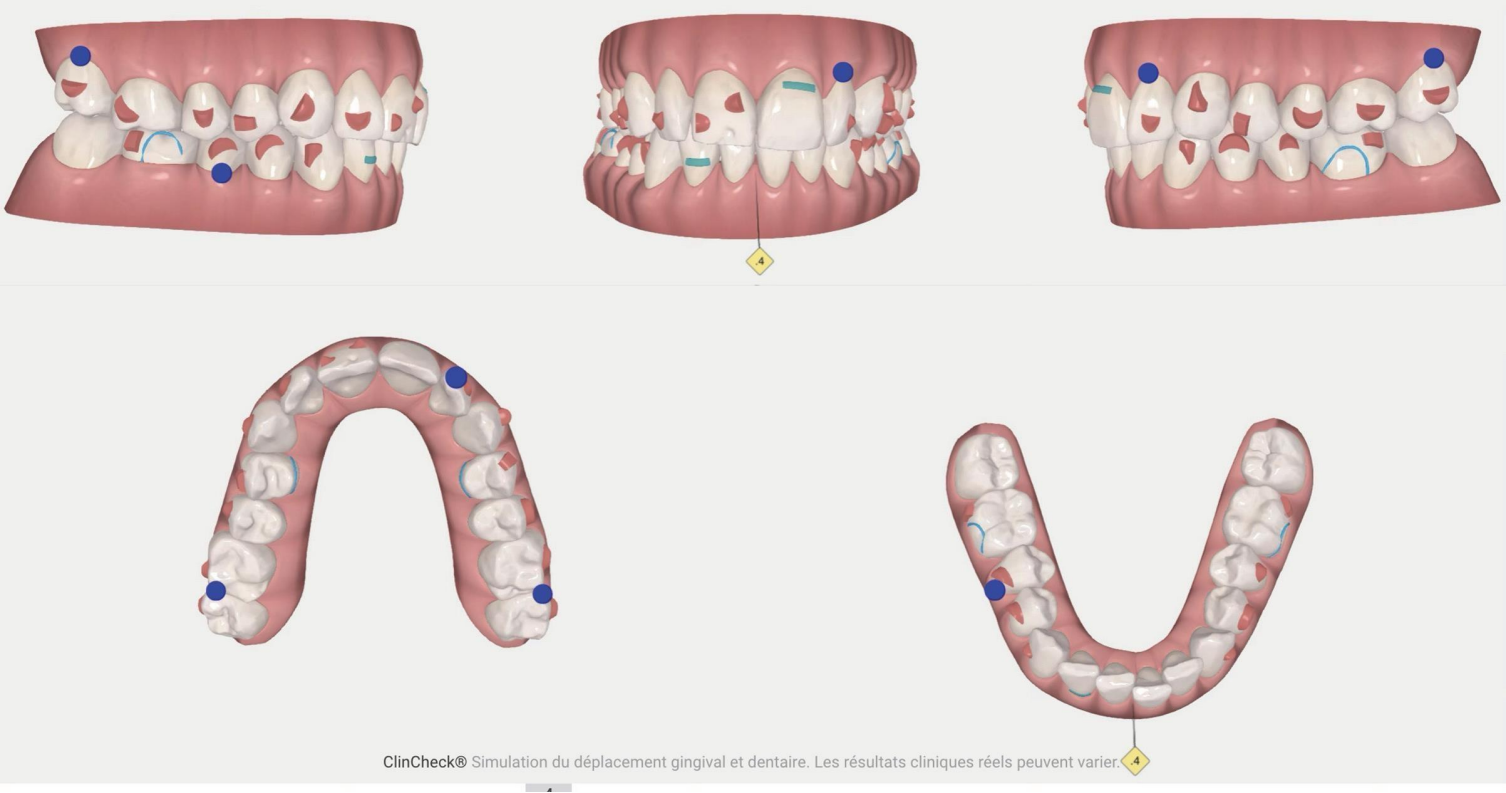
Attachments and auxiliaries’ placement at aligners *n*°4.

To ease the correction of the overbite and the mild Class 2 canine, elastics placed from vestibular side of the lower moral and the palatal side of the upper first premolar, were used during the night (3/16 4 oz) with the first set of aligners. The patient had to wear the aligner 22 h/day, and the changing protocol was set at 14 days before placing the new aligner (Figures 4 and 5). Attachments were placed at aligner number 4, giving the patient an easy and soft start of the treatment with small forces. This protocol eases the adaptation of the patient to his aligners.

**Figure 6:**
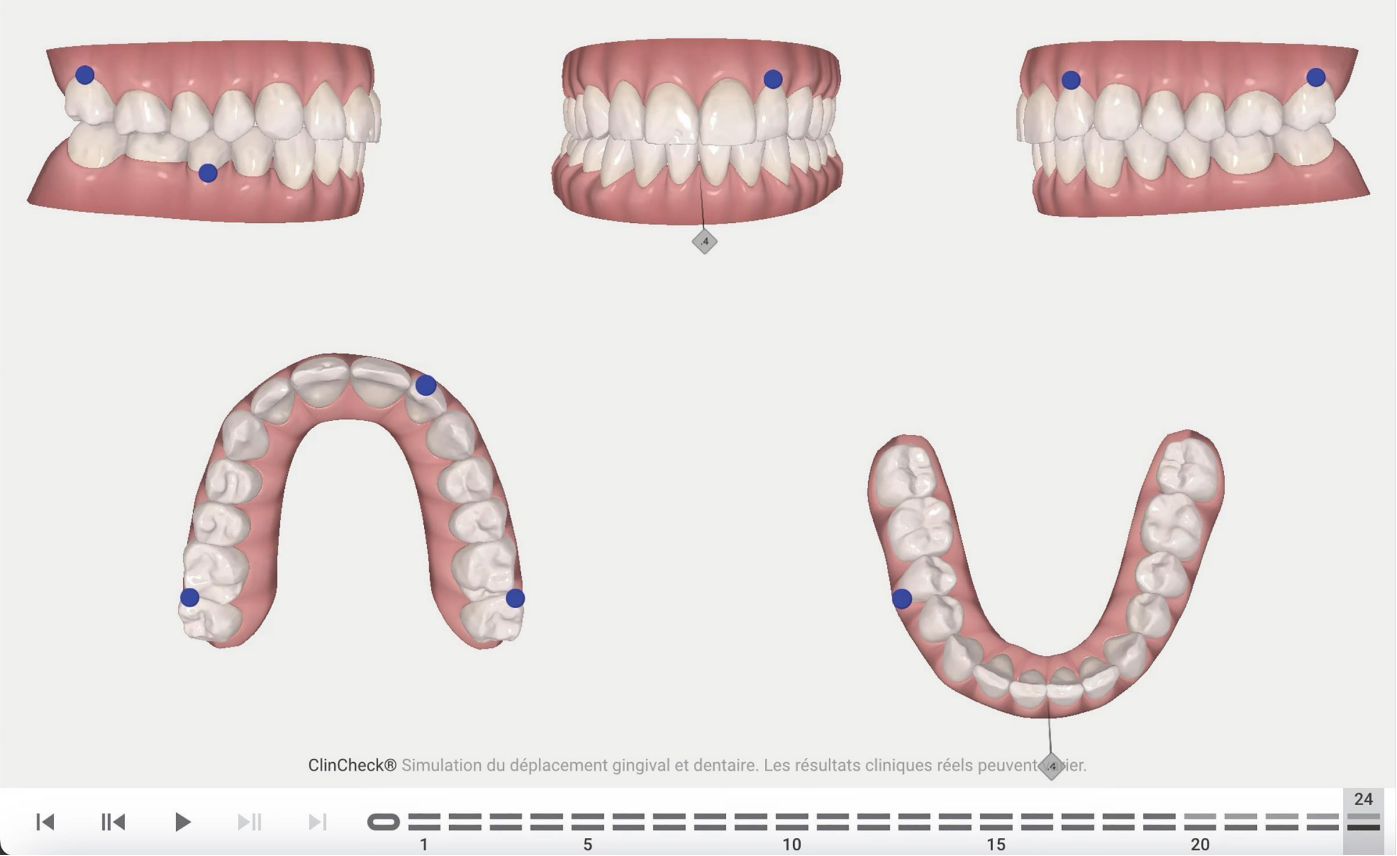
Second phase Clincheck.

**Figure 7:**
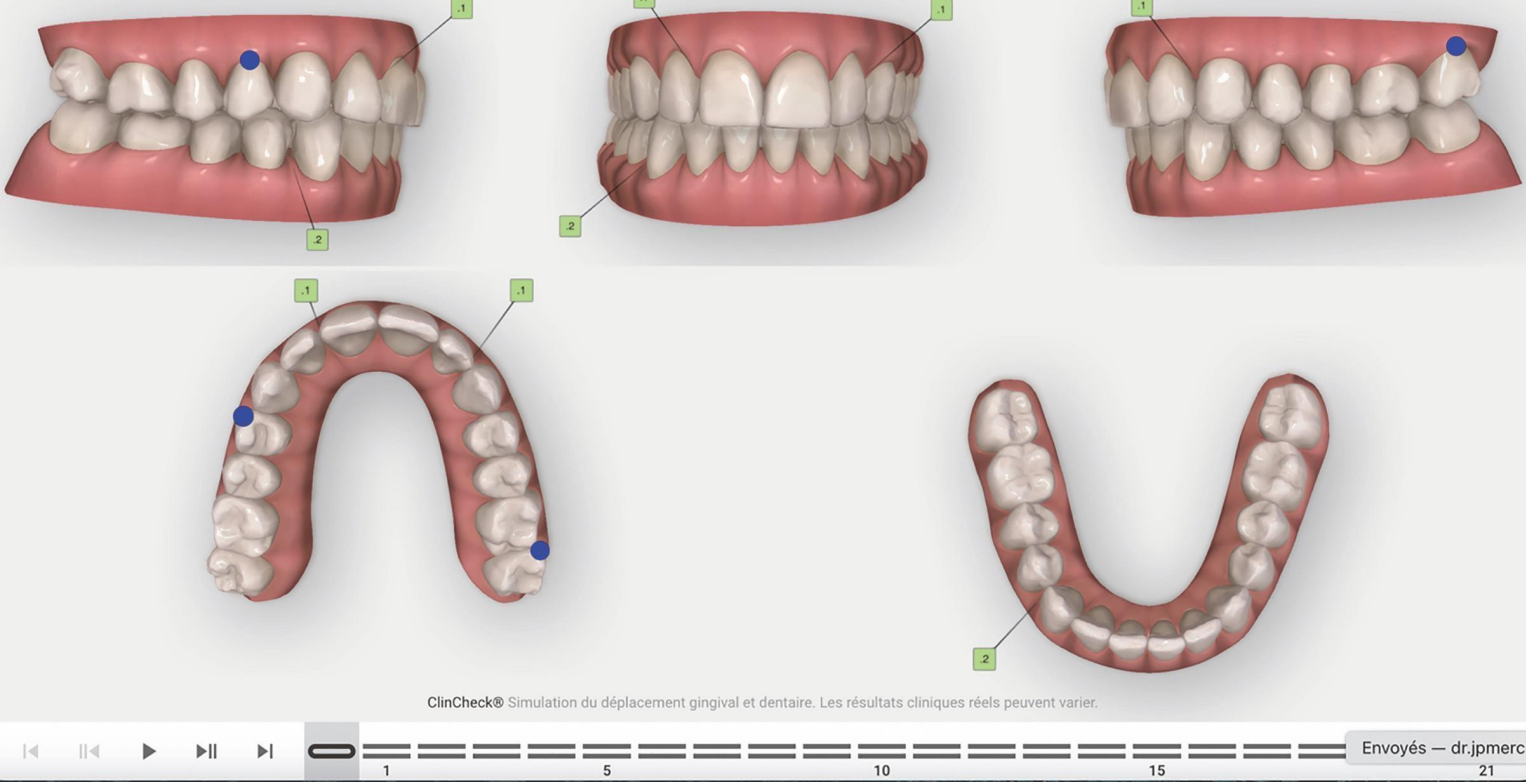
Expected result of the second phase.

**Figure 8:**
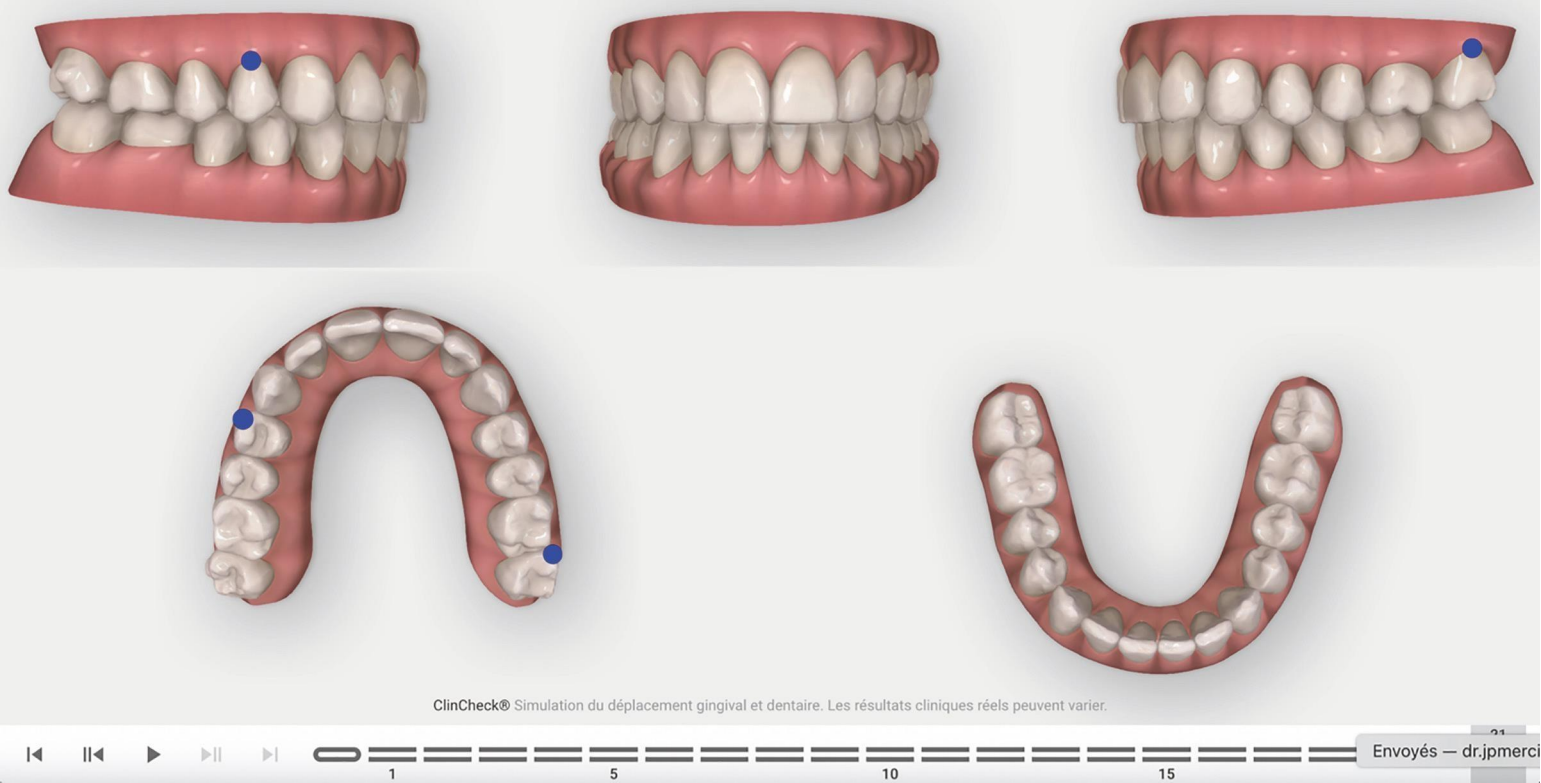
Second Clincheck staging.

**Figure 9:**
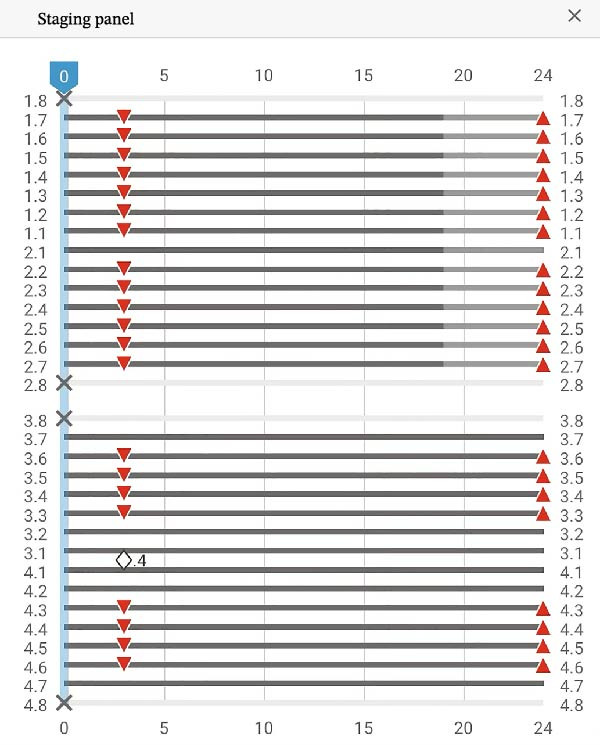
Expected results before the refinement phase.

**Figure 10:**
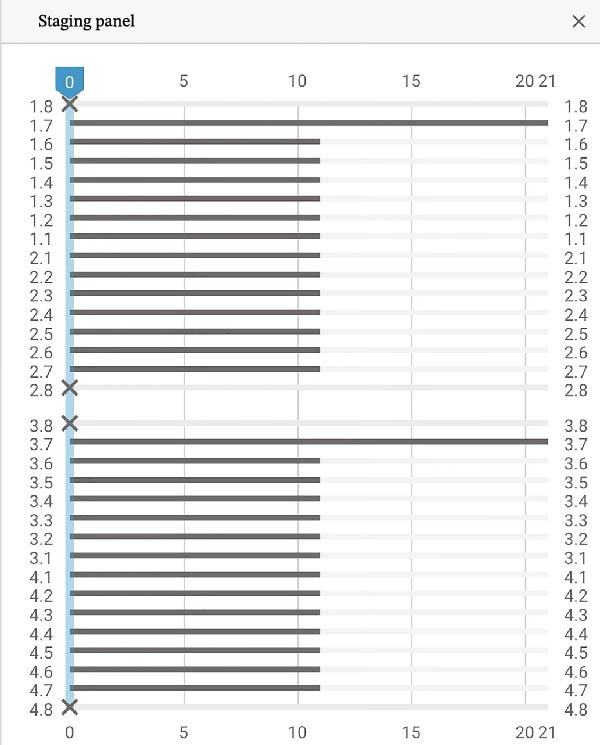
First Clincheck staging.

**Figure 11:**
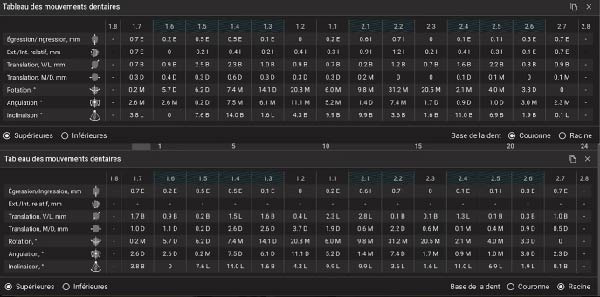
Table of expected tooth movement of the upper arch (crowns and roots).

**Figure 12:**
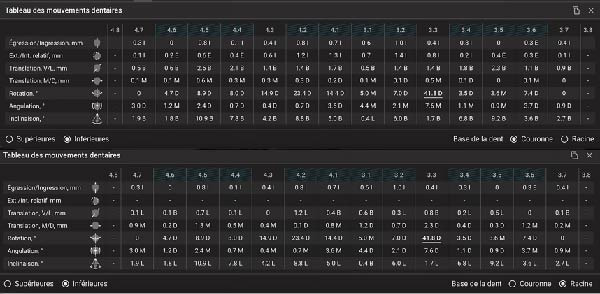
Table of expected tooth movement of the lower arch (crowns and roots).

The second phase involved 21 aligners, this time without attachments as the patient asked for no attachments for esthetic reason. The changing protocol was modified to a weekly change, without modifying the wearing protocol set at 22 h/day (Figures 6–8). As the patient was managed correctly at the periodontal level, we decide to maintain the standard velocity of tooth movement during both phase of the treatment (0.25 mm/aligners). To mitigate any excess of vestibular proclination of the incisors, minor interproximal reduction of the lower incisors was employed and realized before the aligner *n*°4 (Figures 4, 5, 9, and 10). Expected movement of crowns and roots was assessed in the Figures 11 and 12.

To monitor correctly the patient, appointment every 2 month was done to check the good adaptation and the correct evolution of the treatment and tooth movement. No compliance monitoring was used during the treatment.

Upon completion of orthodontic adjustments, fixed custom‐made retainers were placed from premolar to premolar in the upper arch and from canine to canine in the lower arch, strategically designed to avoid intrusion into interdental spaces, thus preserving oral hygiene and avoiding possible relapse.

**Figure 13:**
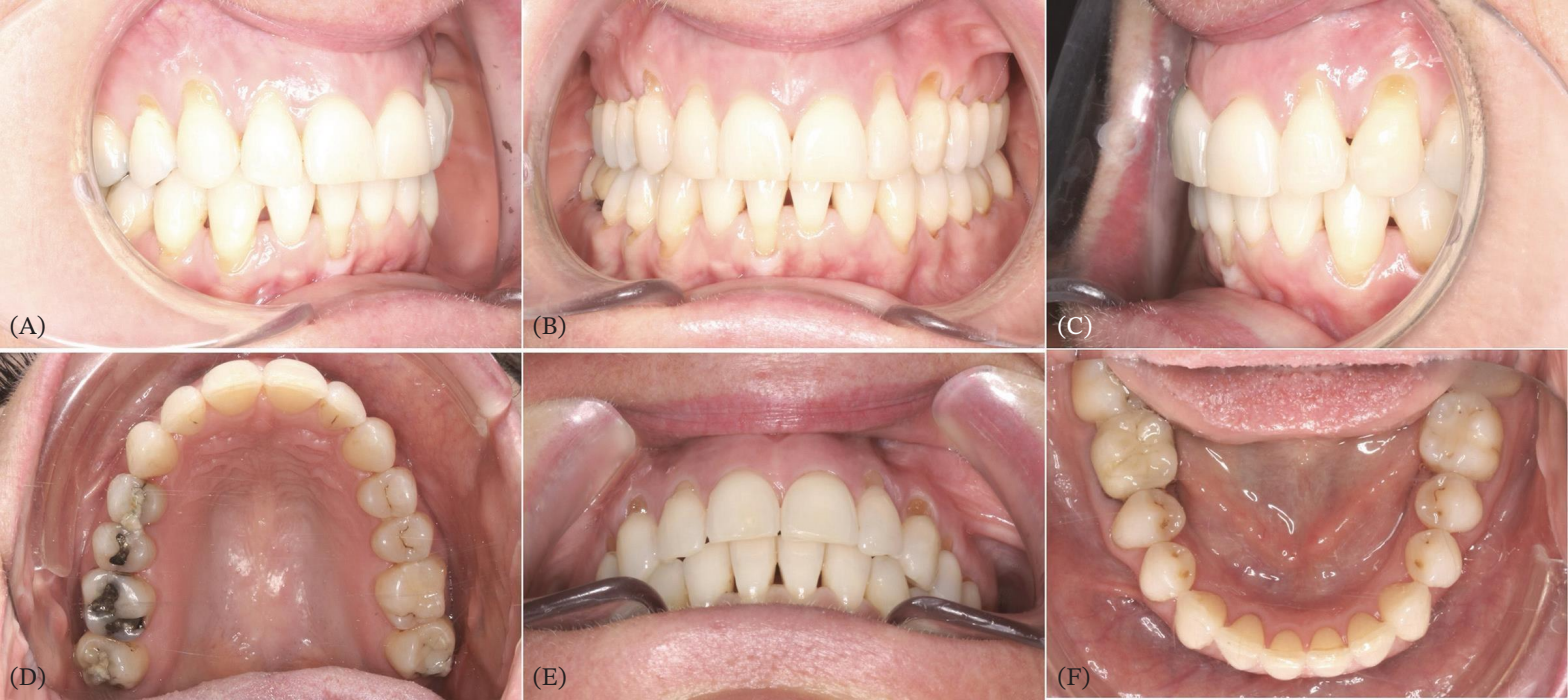
Posttreatment intraoral photo. Subfigures (A–C, E) show the patient’s occlusion after orthodontic treatment. Subfigures (D, F) show the arches.

**Figure 14:**
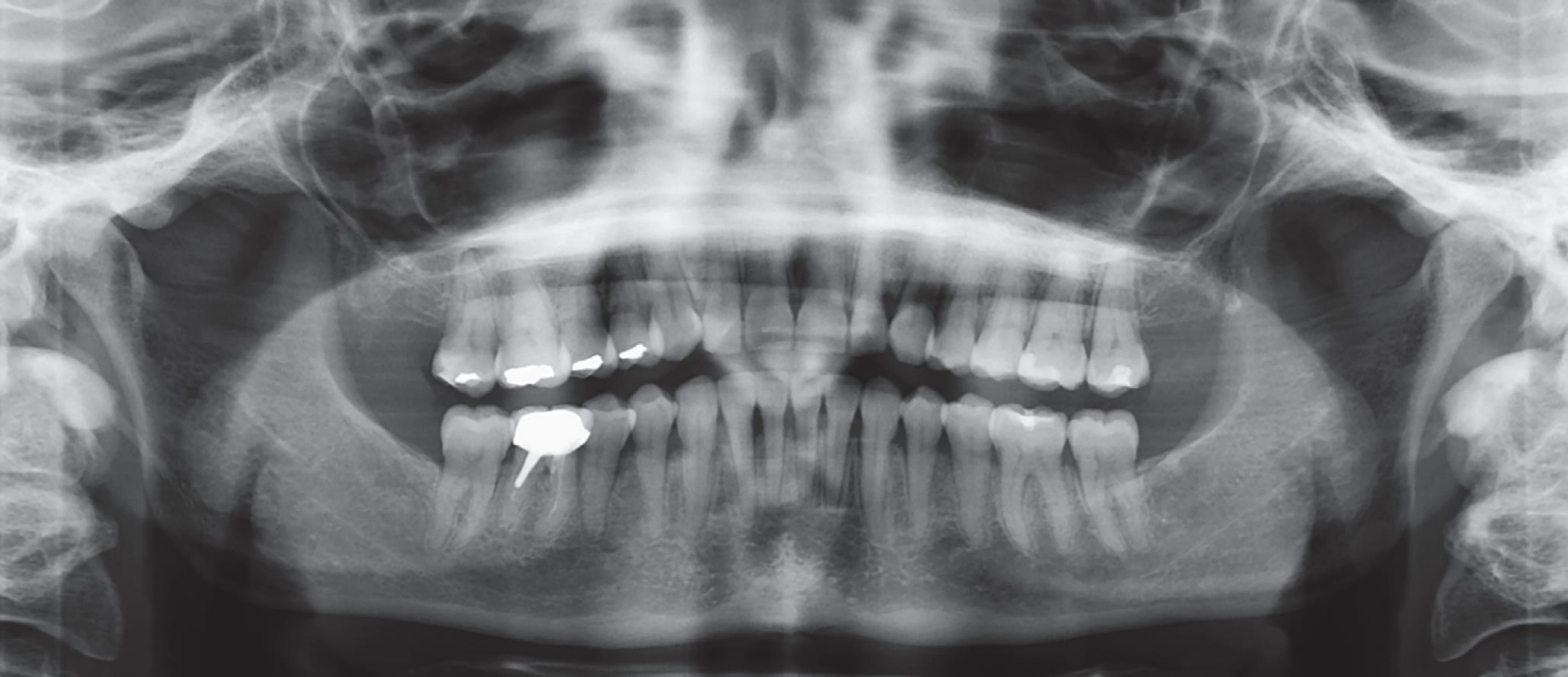
Posttreatment panoramic X‐ray.

After the removal of the attachments and the placement of the retainers, a periodontal surgical therapy was performed. Three successive grafts were made: the first from Teeth 11 to 15, the second from 21 to 25, and the third from 31 to 41. The patient subsequently achieved a harmonized and esthetically pleasing smile characterized by incisors perfectly aligned along the smile arc, with no evidence of additional postorthodontic bone loss (Figures 13 and 14).

Clinical examination and descriptive digital superimposition suggested that the orthodontic treatment objectives were largely met, with tooth positions appearing clinically acceptable at the end of treatment.

**Figure 15:**
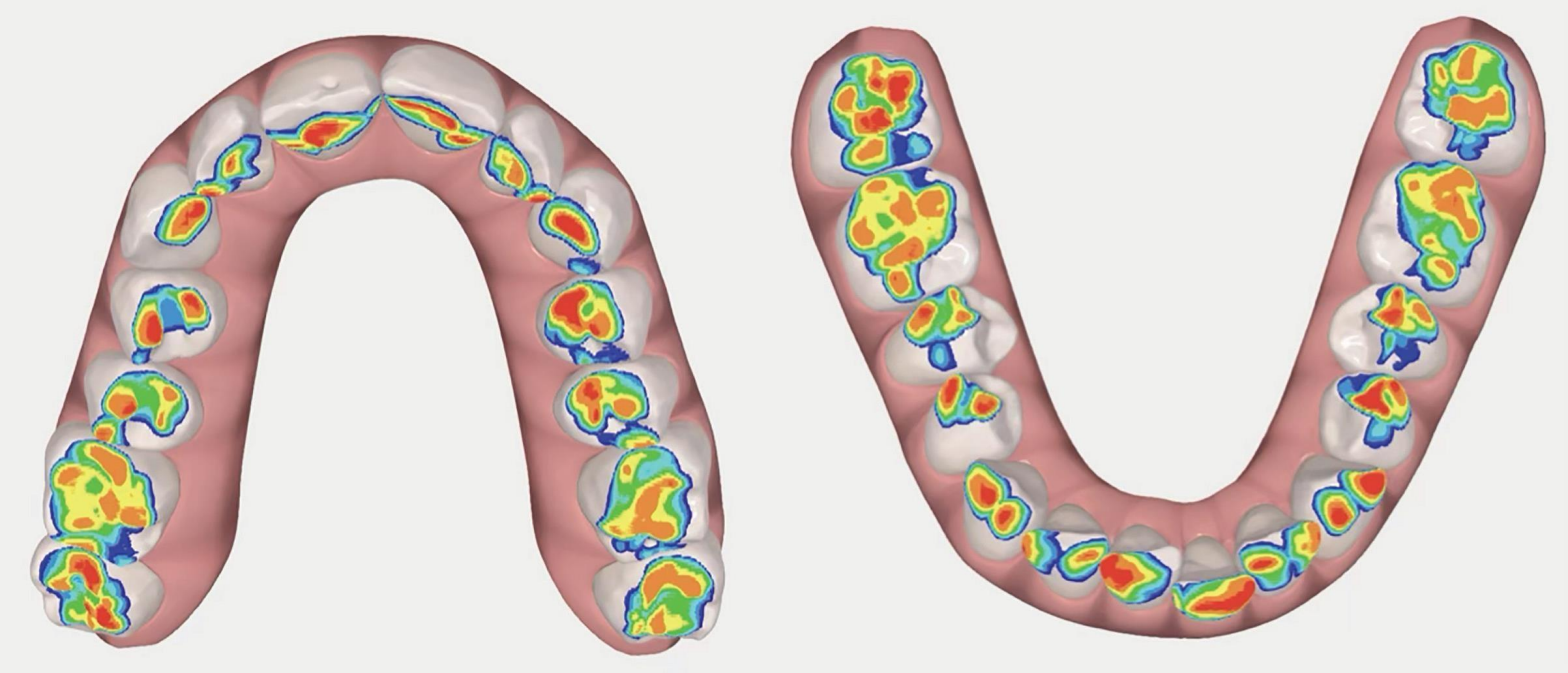
Initial occlusal heat map.

**Figure 16:**
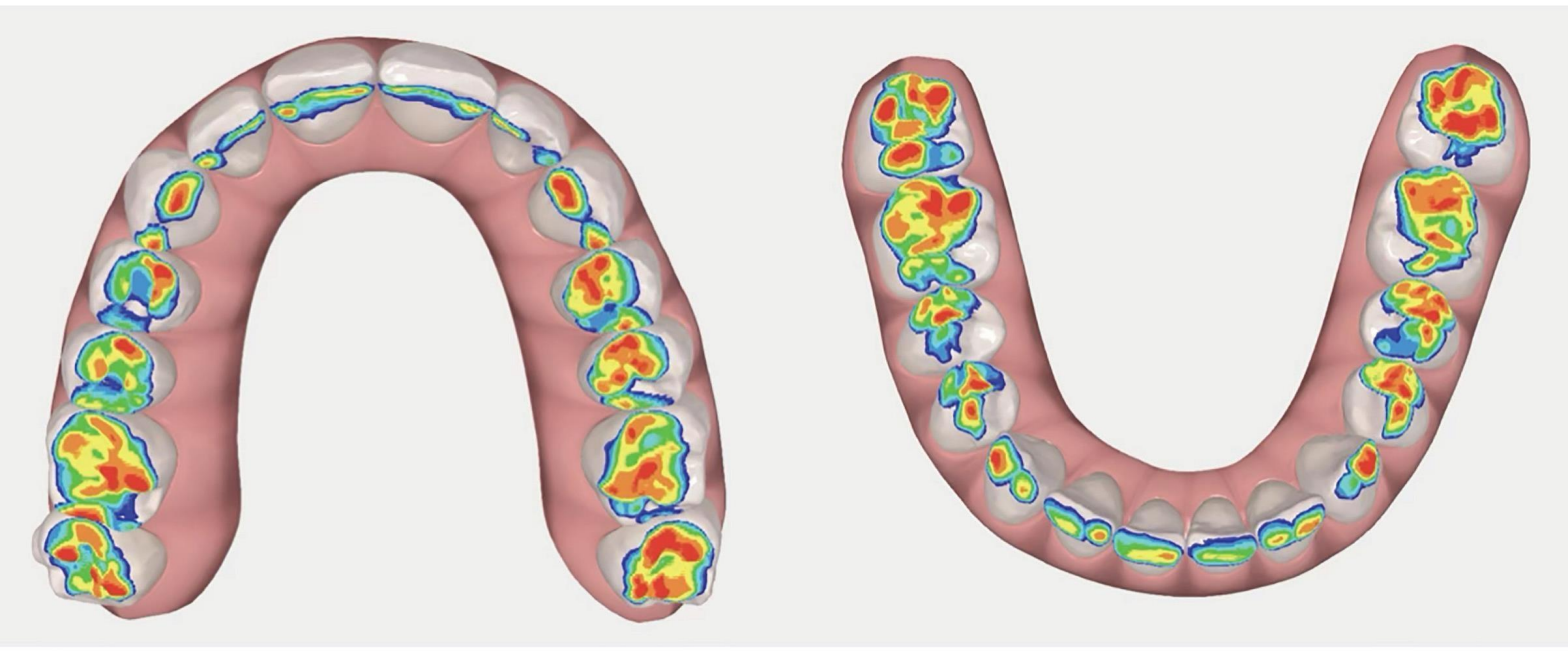
Final occlusal heat map.

To illustrate changes in occlusal contacts, occlusal recordings obtained before and after aligner treatment are presented in Figures 15 and 16. These images descriptively show a redistribution of occlusal contacts, with reduced anterior contact involvement and a more homogeneous posterior contact pattern after treatment. Occlusal heat maps were used for qualitative visualization purposes only.

**Figure 17:**
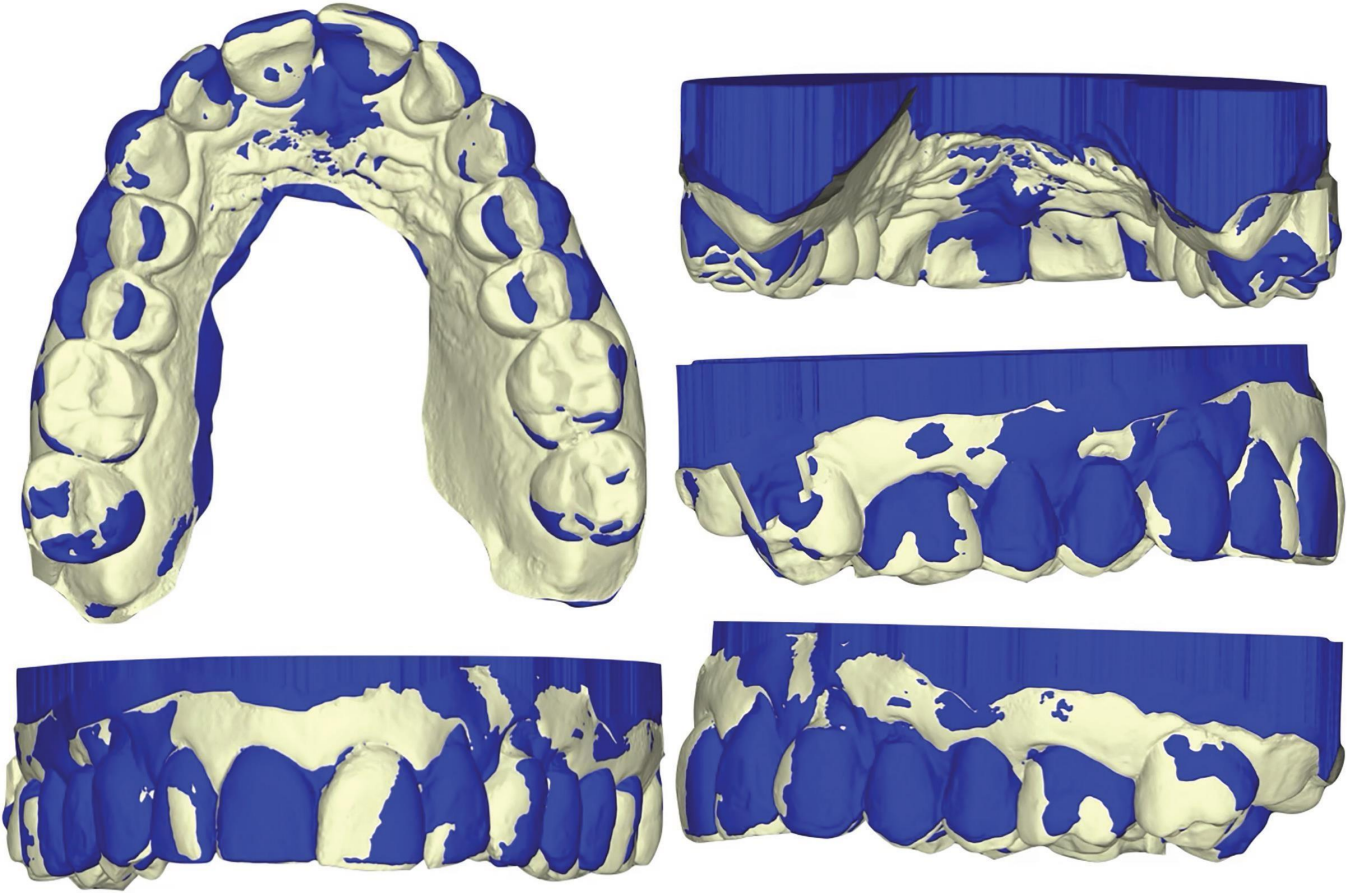
Cephalometric superimposition pre‐ and posttreatment.

**Figure 18:**
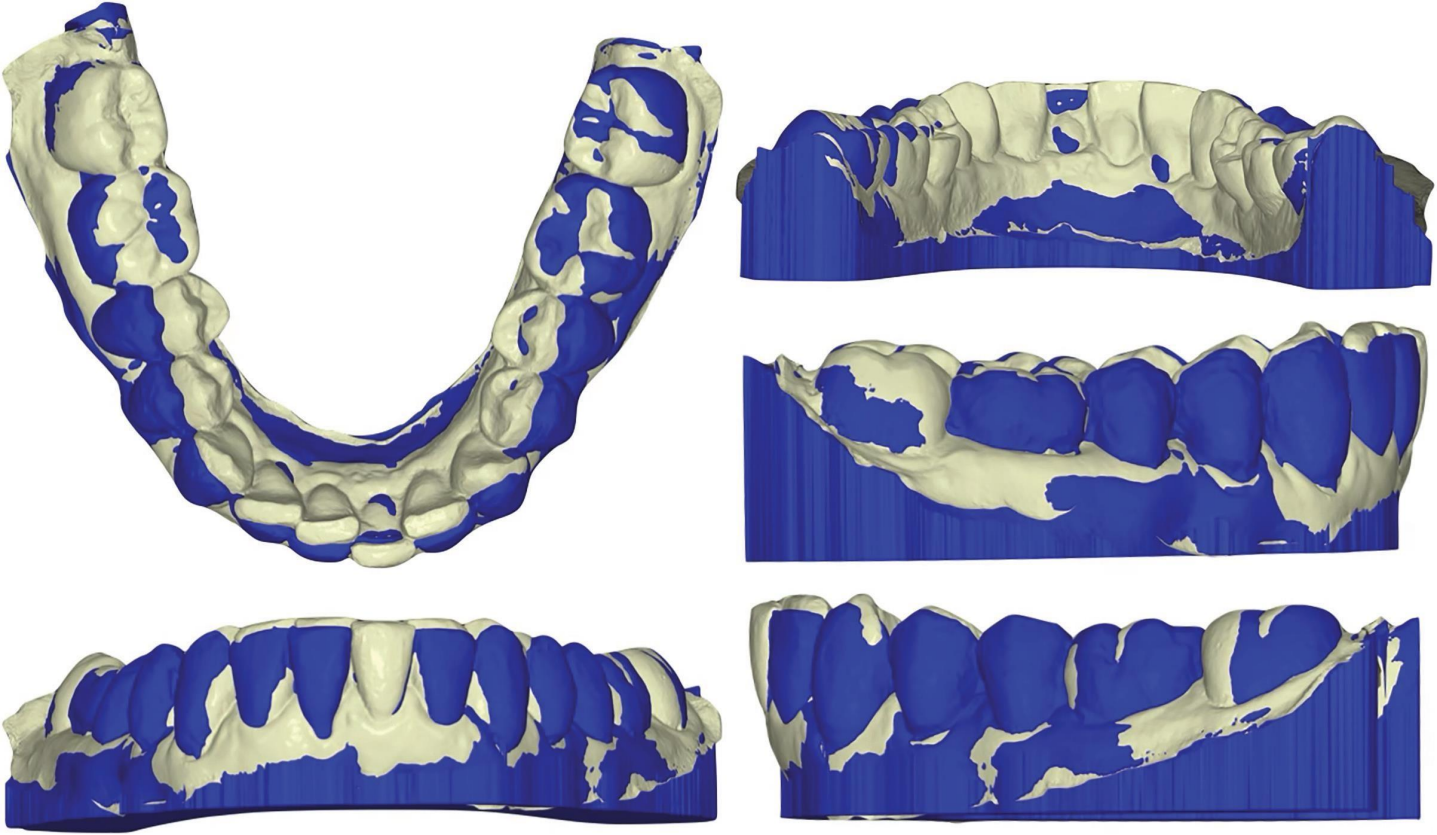
Superimposition of the upper arch pre‐ and posttreatment.

**Figure 19:**
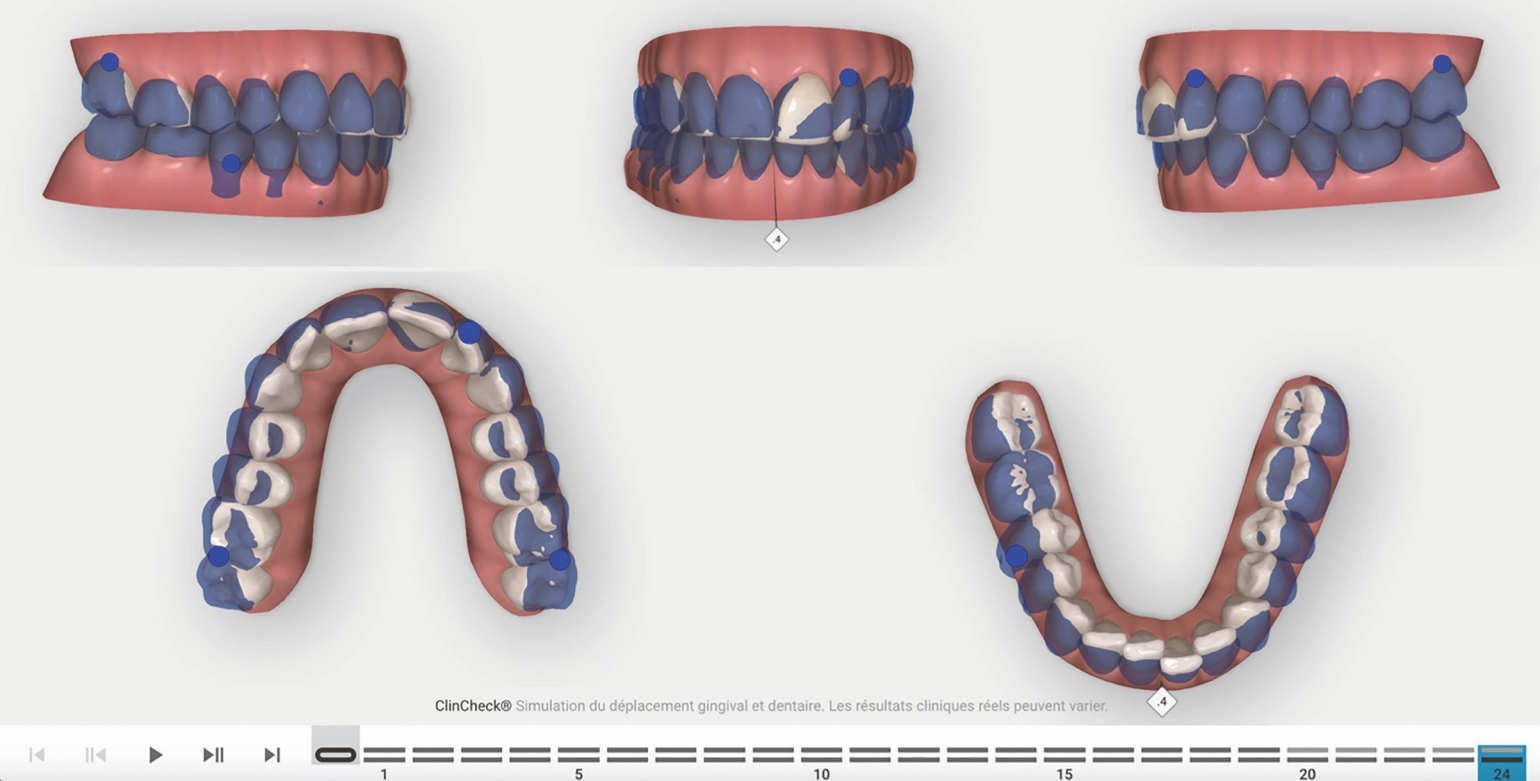
Superimposition of the lower arch pre‐ and posttreatment.

**Figure 20:**
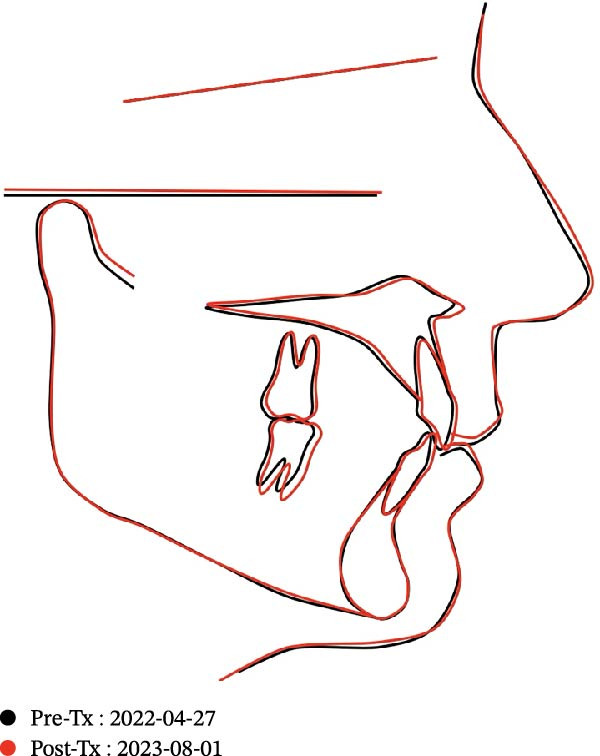
Clin check planned tooth movement results.

Cephalometric superimposition (Figure 17) and intraoral scan superimpositions (Figures  18–20) illustrate changes in the upper and lower arches before and after treatment. Digital superimpositions were performed using a surface‐based best‐fit alignment, primarily relying on the palatal rugae, which are considered relatively stable anatomical structures in adult patients. Posterior teeth were used as secondary reference areas. This approach was employed for descriptive visualization purposes only and not as a quantitative assessment of tooth movement accuracy [16].

In the upper arch, transverse changes in the premolar region, anterior alignment, rotations, and changes in crown inclination were observed. The treatment appeared to maintain the roots within the alveolar housing with just a small amount of proclination of the incisors.

The digital setup was used as a visual planning tool rather than as a biological reference standard. Clinical examination and digital superimposition suggested that the achieved tooth movements were consistent with the treatment objectives.

Cephalometric analysis indicated slight proclination of the upper and lower incisors, consistent with the correction of the mild crowding present before treatment. Skeletal measurements remained unchanged, as the patient was an adult with no remaining growth potential (Table 2).

**Table 2:** Cephalometric analysis before and after treatment.

Parameters	Before	After
SNA	80.65	79.86
SNB	76.87	76.29
ANB	3.78	3.57
Bjork sum	389.4	388.63
Wits appraisal	1.15	1.13
U1 to FH	101.0	106.43
U1 to SN	92.99	97.45
IMPA	97.61	103.92
Interincisal angle	140.01	130.01
U1 to NA (mm)	2.9	4.82
U1 to NA (°)	12.34	17.59
L1 to NB (mm)	5.13	6.55
L1 to NB (°)	23.86	28.83

## 3. Periodontal Treatment Protocol

Composite restorations were performed at the maximal coverage line to recreate the cemento‐enamel junction (CEJ) [17].

**Figure 21:**
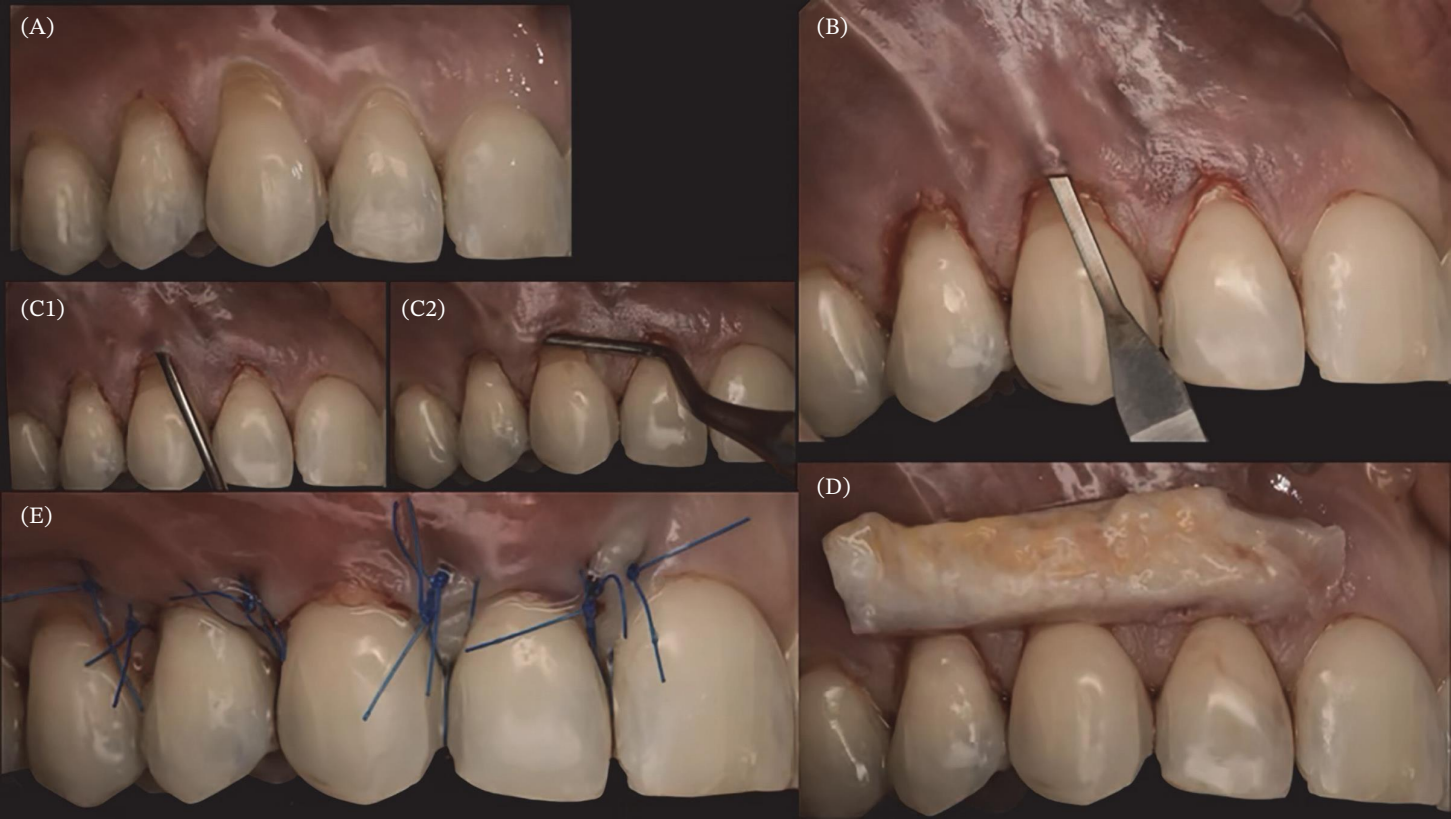
Periodontal plastic surgery with coronally advanced tunnel in Sector 1. (A) Placement of composite restorations at the maximum coverage line. (B) Flap elevation using tunneling elevators from teeth 11–15. (C, C1,C2) Dissection beyond the mucogingival line with an MJK spoon blade. (D) Lamina propria connective tissue graft from teeth 12–14. (E) Sutures performed with 5.0 nylon monofilament thread (Arago).

**Figure 22:**
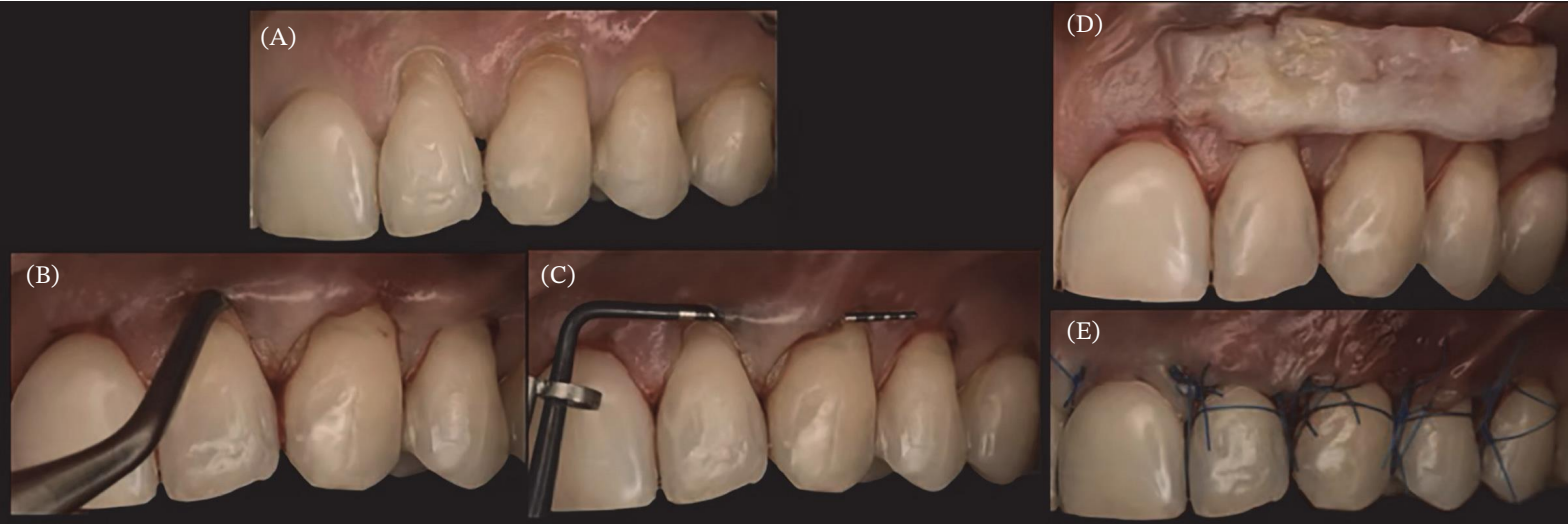
Periodontal plastic surgery with coronally advanced tunnel in Sector 2. (A) Placement of composite restorations at the maximum coverage line. (B) Flap elevation using tunneling elevators from teeth 21–25. (C) Verification of the tunnel using a periodontal probe. (D) Lamina propria connective tissue graft from teeth 22–24. (E) Sutures performed with 5.0 nylon monofilament thread (Arago).

For periodontal plastic surgery targeting coverage, a modified tunnel technique [18] with a coronally advanced buried connective tissue graft was employed on the maxilla. All procedures were performed by a single operator (Figures 21 and 22). After administering local anesthesia (articaine 1:100,000) buccally and palatally, intrasulcular incisions were made from Teeth 11 to 15 and 21 to 25, preserving interdental papillae (MJK BW002). The flap was raised to the mucogingival junction using tunnel elevators (Deppeler) and extended apically with MJK Spoon blades. Flap flexibility and elevation were confirmed with a PCPUNC15 periodontal probe.

A palatal epithelium‐connective tissue graft was harvested and de‐epithelialized with a #15 blade. It was inserted into the tunnel through the deepest recession (canine) and positioned in Sectors 1 (Teeth 12–14) and 2 (Teeth 22–24), then secured with sutures. The flap was coronally sutured with “double cross” [19] and/or “belt‐and‐braces” [20] techniques (Figures 21 and 22).

**Figure 23:**
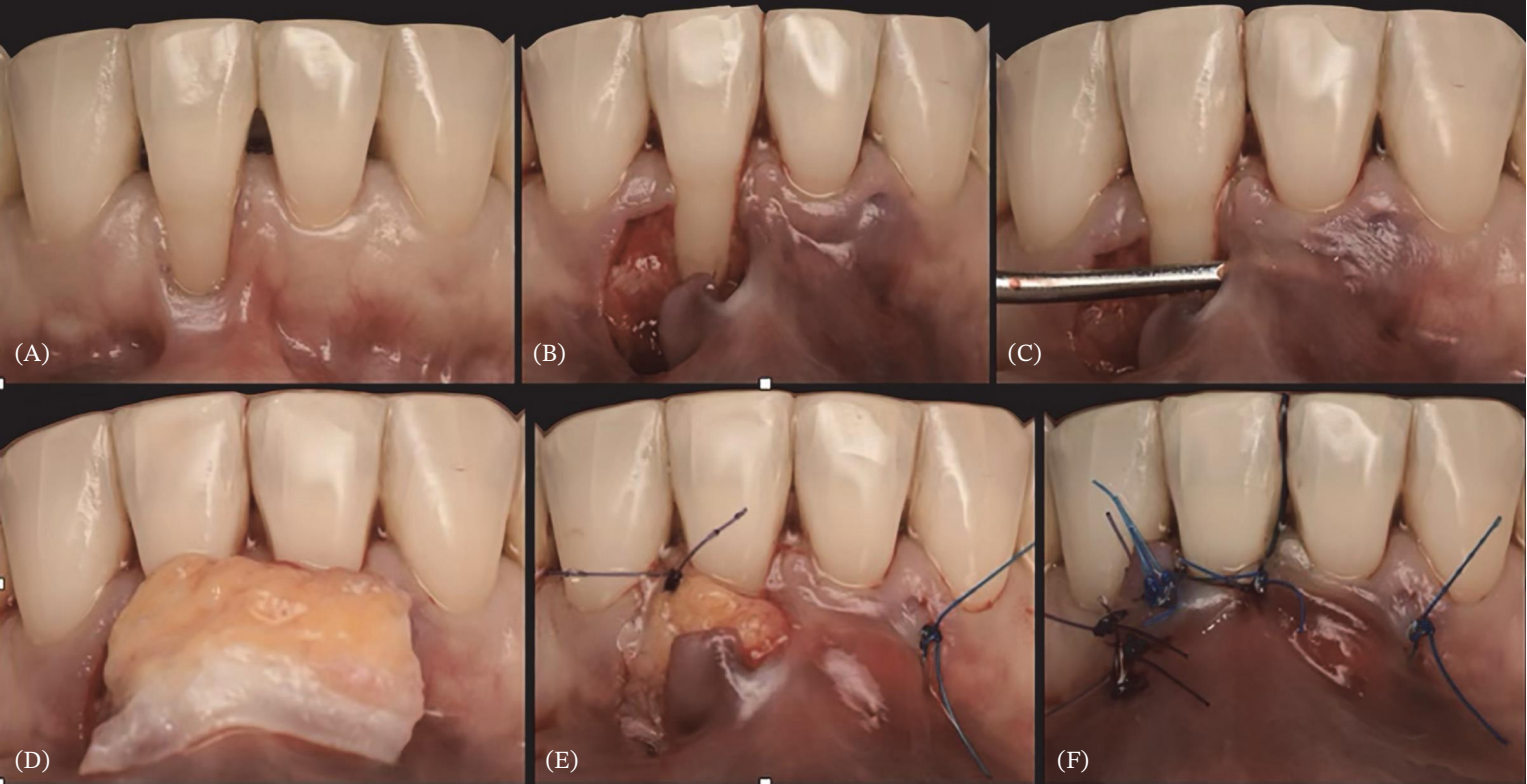
Periodontal plastic surgery with a tunneled and coronally advanced flap on Tooth 41. (A) Initial situation: RT2 recession, absence of keratinized gingiva, frenulum pulling on the marginal gingiva and opening the sulcus. (B) Horizontal and vertical incisions to create the surgical papilla. (C) Tunnelization. (D) Lamina propria connective tissue graft from teeth 31 to 41. (E) Suturing of the graft to the tunnel and the de‐epithelialized anatomical papilla. (F) Suturing of the flap with suspensory stitches and of the releasing incision with simple sutures.

For recession on mandibular Tooth 41, a tunneled coronally advanced flap (T‐CAF) was used [21]. Para‐apical anesthesia (articaine 1:100,000) was administered, followed by intrasulcular incisions preserving the mesial papilla and a distal horizontal incision to form a surgical papilla. The flap was dissected in partial thickness and extended beyond the mucogingival line for tension‐free elevation. A palatal epithelium‐connective tissue graft was harvested and inserted into the tunnel via a releasing incision, secured with mattress and suspensory sutures (Figures 23).

Postoperative care:

• Antibiotics: Amoxicillin (2 g/day for 1 week, starting preoperatively),
• Corticosteroids: Prednisolone (60 mg/day for 3 days, beginning surgery day),
• Analgesics: Paracetamol (1 g every 6 h for 3 days),
• Chlorhexidine gel and mouthwash.

Patients were advised to avoid brushing the surgical area until suture removal 2 weeks later. Palatal donor sites were protected with a palatal plate.

**Figure 24:**
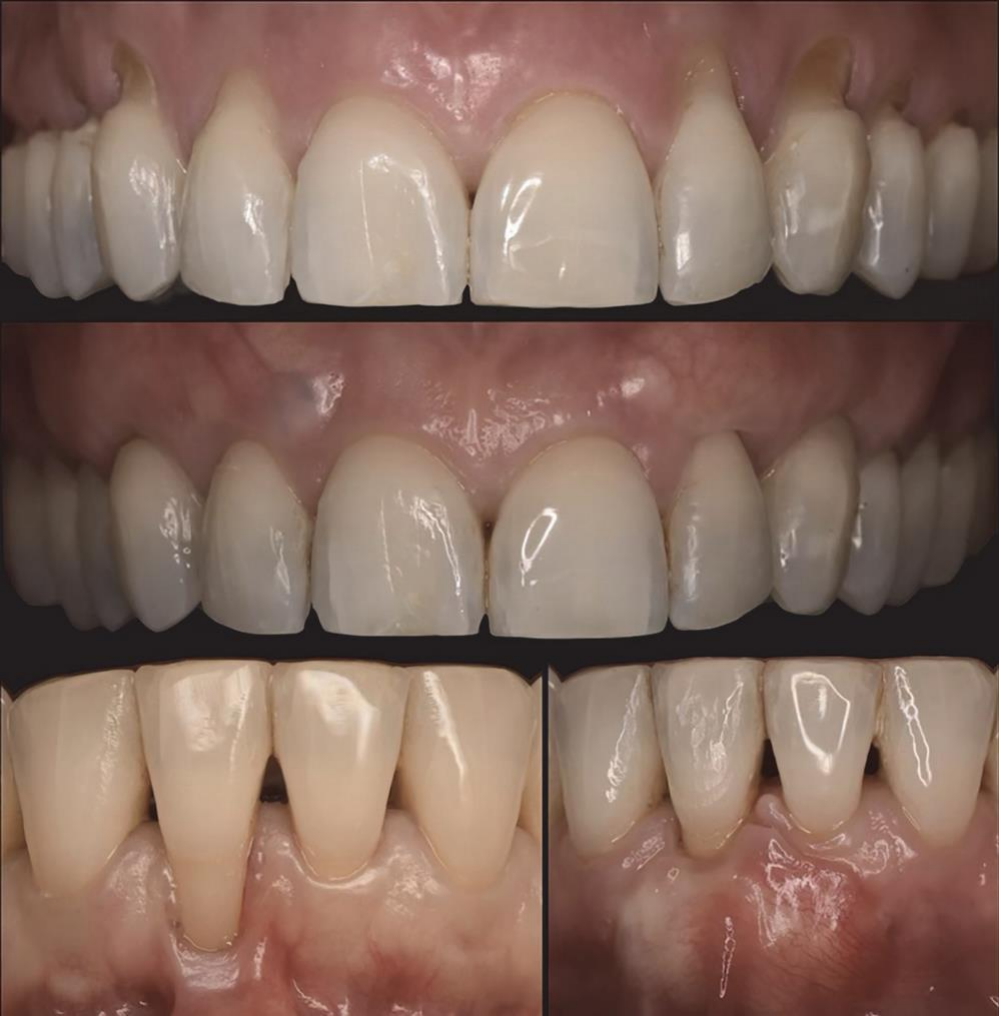
Initial situation and at 1‐year postoperative.

**Figure 25:**
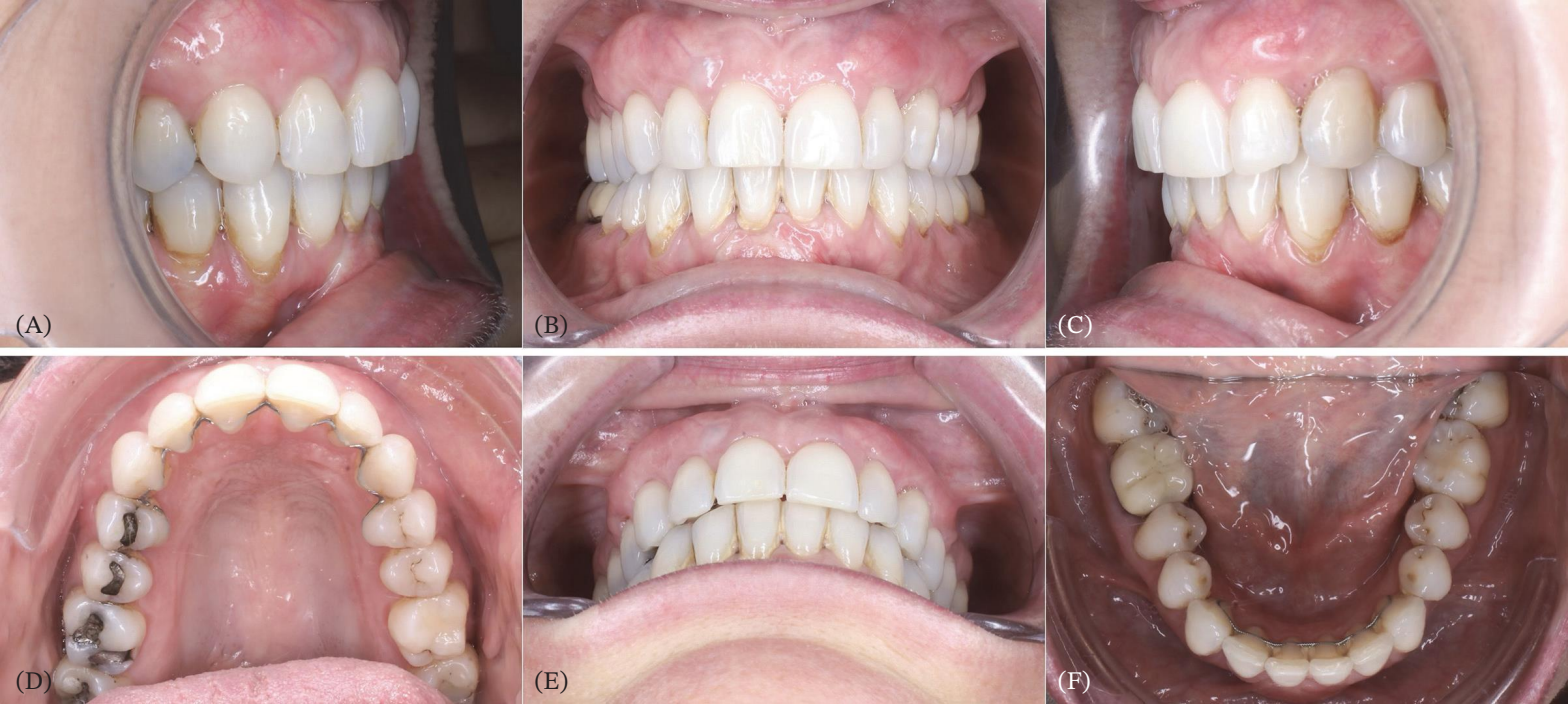
Intraoral photograph at 1‐year postperiodontal treatment. Subfigures (A–C, E) show the patient’s occlusion after orthodontic treatment and gingival grafting. Subfigures (D, F) display the arches.

**Figure 26:**
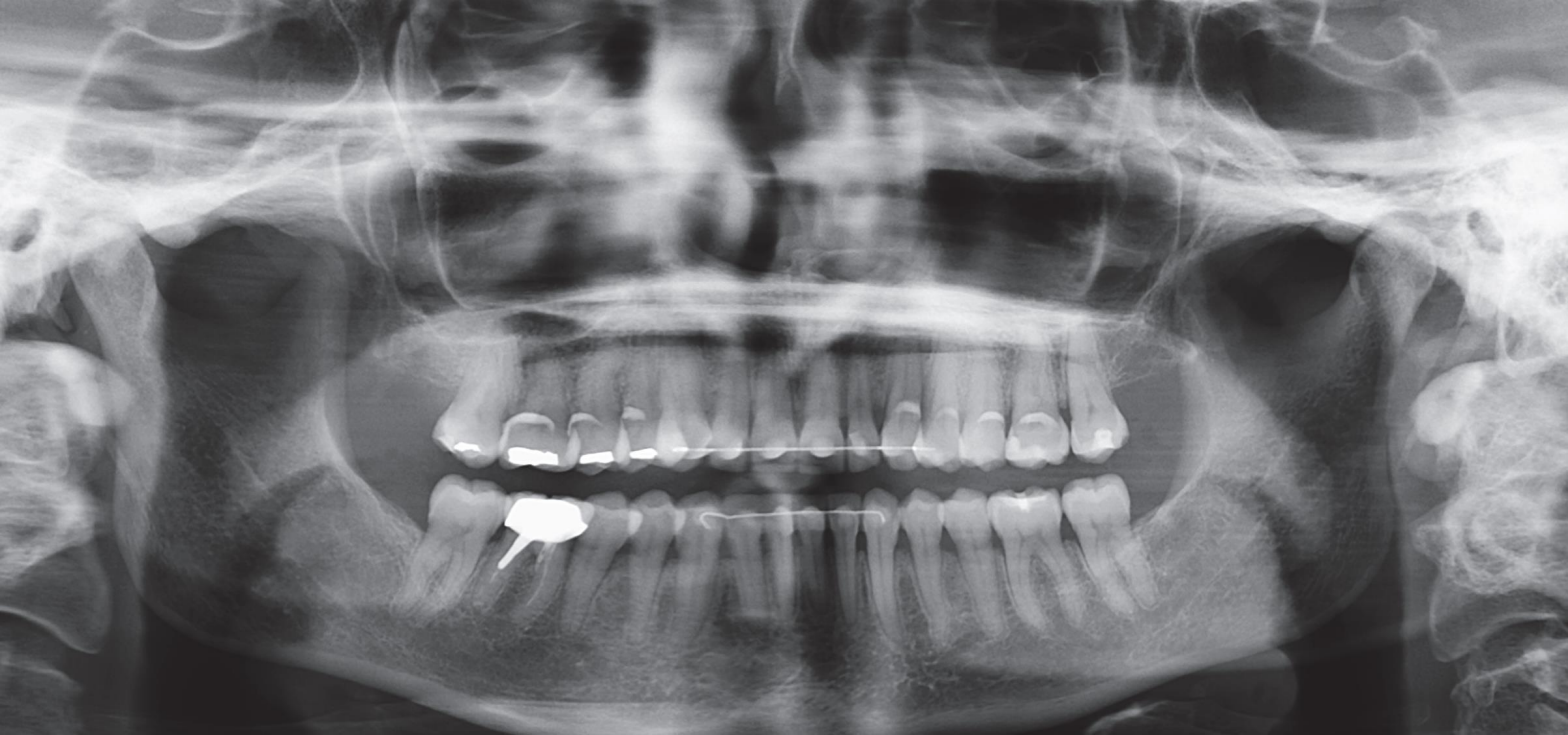
Facial photographs pre‐ and postorthodontic treatment, as well as postperiodontal treatment.

Post orthodontic treatment at 1 year retention and post periodontal treatment results are showed in Figures 24–26.

Throughout the treatment period, no contingency planning was required, as the patient adhered to the recommendations provided by both the orthodontist and the periodontist. Following oral hygiene instruction and motivation during the initial nonsurgical periodontal therapy phase, a baseline periodontal evaluation was performed by the treating periodontist prior to orthodontic treatment. Plaque index was below 20% and bleeding on probing was below 10%, indicating good plaque control and limited gingival inflammation at baseline. Periodontal assessment did not reveal any clinical signs of active periodontitis. However, standardized site‐specific periodontal measurements, including probing depths, clinical attachment levels, and recession measurements, were not systematically recorded at baseline.

**Figure 27:**
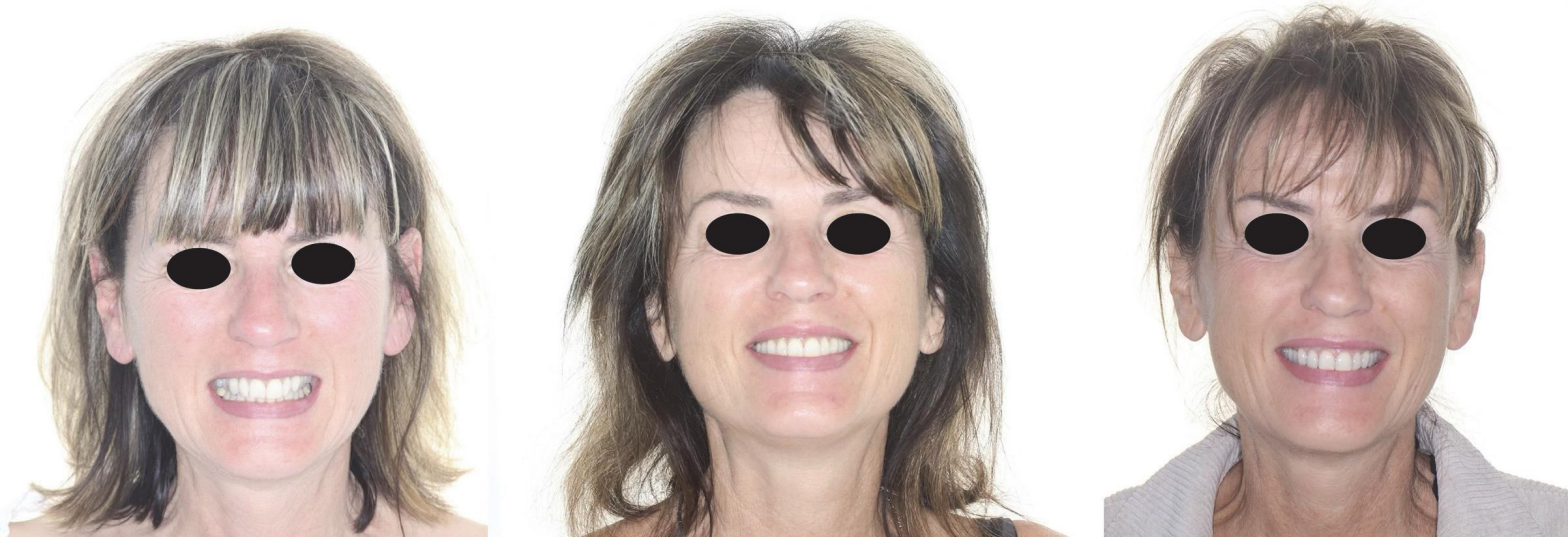
Panoramic radiograph 1‐year posttreatment.

From a clinical perspective, orthodontic treatment was associated with improved oral hygiene conditions. Compared with the beginning of treatment, fewer plaque‐retentive areas were observed in the lower arch, allowing easier access for optimal oral hygiene maintenance. Bone levels appeared stable and clinical conditions remained unchanged at the 1‐year follow‐up (Figures 25–27). From the patient’s perspective, satisfaction with both esthetic and functional outcomes was reported. The patient felt more confident when smiling and reported that tooth cleaning was easier, particularly in the lower incisor region where crowding had been present before treatment.

## 4. Discussion

Clear aligners have emerged as a viable option in orthodontic treatment, particularly for repositioning lower incisor roots toward a more favorable position within the alveolar housing, which may be relevant in the management of GR and gingival thinning [22–24].

Several studies have reported that aligners can achieve lingual root torque of lower incisors, whereas torque control of maxillary incisors remains less predictable. The distinct anatomical characteristics of lower incisor roots, which are generally shorter and more slender, may partly explain these differences. In cases classified as Miller Class I or Cairo RT1 recessions, orthodontic intervention with aligners alone has been reported to reduce recession severity and, in some situations, limit the need for surgical root coverage [25].

In more advanced recessions, such as Miller Class II or Cairo RT2 lesions, aligner therapy may contribute to reducing the extent of recession, particularly in the mesiodistal dimension, by repositioning roots within the alveolar envelope. Bucur et al. [26] reported changes in recession severity following aligner treatment in patients presenting RT1 and RT2 lesions, with a tendency toward reduced gingival thinning and improved periodontal conditions.

According to recent literature, the novelty of such approaches lies not in the use of aligners as an alternative to mucogingival surgery, but rather as a complementary strategy aimed at optimizing tooth position prior to or in conjunction with periodontal surgical management. Retrospective studies and clinical case reports have described improvements in mild to moderate recessions (RT1–RT2) following aligner therapy [26, 27].

Furthermore, comparative studies suggest that combined orthodontic and periodontal approaches may provide higher root coverage rates for RT2 defects compared with isolated surgical procedures, supporting the value of a multidisciplinary treatment strategy [28].

Accurate classification of GRs is essential for diagnosis and treatment planning. Recent methodological literature emphasizes the advantages of the Cairo classification (RT1–RT3) over the Miller classification in characterizing recession severity and facilitating comparison of therapeutic outcomes. This standardized approach improves the reproducibility of clinical reports evaluating orthodontic interventions in patients with GR [29].

The interdisciplinary management described in the present case is consistent with previously published reports, such as that of Ohira et al. [30], which highlighted close collaboration between orthodontists and periodontists combining aligner therapy with periodontal surgery.

### 4.1. Biomechanical and Clinical Considerations

Despite technological advances, the use of clear aligners in periodontally compromised patients presents several biomechanical and clinical challenges. Precise treatment planning is required to control root positioning while minimizing the risk of adverse periodontal effects. Although CBCT–based planning systems may enhance root torque assessment, effective root control with aligners often depends on case‐specific modifications of aligner design and treatment protocols, including attachments, power ridge features, trim line alterations, reduced velocity of tooth movement, and occlusal equilibration. The scientific evidence supporting each of these strategies remains limited and continues to evolve [1, 31–38].

Orthodontic treatment may contribute to periodontal stability by repositioning roots within the alveolar housing and promoting physiological bone remodeling mediated by periodontal ligament activity. Root movement centered within the alveolar process has been associated with a lower risk of GR, whereas vestibular displacement beyond the bony envelope may favor dehiscence and recession [39].

When adequately controlled, bodily tooth movement may facilitate soft tissue adaptation and create more favorable conditions for subsequent mucogingival surgery [40, 41].

In this context, orthodontic tooth movement has been proposed as an adjunctive tool in periodontal and osseous remodeling by stimulating bone turnover through controlled mechanical loading. Experimental and clinical studies suggest that such movement may improve the periodontal environment; however, orthodontic treatment should not be considered a regenerative therapy per se. Rather, its role is adjunctive, contributing to anatomical and biological conditions that may support periodontal healing [40–42].

Gingival biotype plays a critical role in treatment outcomes, as thin biotypes and narrow buccal bone plates are associated with a higher risk of recession. Careful planning, application of light orthodontic forces, and close periodontal monitoring are therefore essential. Importantly, orthodontic tooth movement itself has been identified as a potential risk factor for GR, particularly in patients with thin periodontal phenotypes or when excessive incisor proclination and movement beyond the alveolar envelope occur [43, 44].

Management of interdental black triangles represents an additional challenge in periodontal patients. These defects are multifactorial and influenced by patient age, tooth morphology, tooth movement, root angulation, periodontal status, and gingival biotype [44, 45]. In the present case, black triangle reduction involved nonsurgical periodontal management, interproximal reduction during orthodontic treatment, and subsequent surgical correction [45, 46].

A major limitation of aligner therapy is its reliance on patient compliance, as removable appliances may result in intermittent force application and reduced predictability [47, 48]. In addition, precise torque expression remains challenging, particularly in the anterior region [49].

### 4.2. Strength and Limits of the Mechanics Used in This Case

In this case, CAT was selected to enable controlled tooth movement under low‐force conditions, which is particularly relevant in an adult patient with reduced periodontal support and a thin gingival biotype. Careful staging and a strict wearing protocol were implemented to favor bodily tooth movement and limit uncontrolled tipping, contributing to clinically acceptable root positioning within the alveolar housing. Digital treatment planning prioritized root control rather than crown alignment alone, and the use of optimized attachments, power ridge features, bite ramps, and selective interproximal reduction facilitated space management without increasing orthodontic forces. Close interdisciplinary collaboration between the orthodontist and the periodontist allowed continuous periodontal monitoring and timely adjustments when required.

Several limitations must be acknowledged. Torque expression with aligners remains less predictable and outcomes are highly dependent on patient compliance. Moreover, no CBCT imaging was performed in accordance with ALARA principles, limiting three‐dimensional assessment of root positioning and root resorption. Importantly, the subsequent connective tissue grafting performed after orthodontic treatment represents a confounding factor, as the respective contributions of orthodontic tooth movement and surgical intervention to the observed periodontal improvements cannot be isolated in this single case report.

## 5. Conclusion

This case report illustrates the importance of close interdisciplinary collaboration between orthodontics and periodontics in the management of an adult patient with compromised periodontal support. In this patient, the use of CAT with case‐specific biomechanical adaptations allowed controlled tooth movement while maintaining periodontal stability, followed by periodontal surgical management.

The clinical observations suggest that, when carefully planned and monitored, clear aligners may serve as a supportive orthodontic tool in complex ortho–periodontal situations. However, the findings from this single case cannot be generalized, and the respective contributions of orthodontic and periodontal interventions to the observed outcomes cannot be isolated.

Further clinical studies with standardized periodontal measurements and comparative designs are required to better define the role of aligner‐based orthodontic treatment in periodontally compromised patients.

## Author Contributions

**Waddah Sabouni:** conceptualization, project administration, supervision, validation, writing – review and editing. **Jean-Philippe Mercier:** conceptualization, investigation, project administration, supervision, validation, visualization, writing – original draft, writing – review and editing. **Arthur Brincat:** conceptualization, investigation, project administration, validation, writing – original draft, writing – review and editing.

## Acknowledgments

We confirm that no artificial intelligence software was used in any part of the research or manuscript preparation, including data collection, writing, or editing.

## Funding

No funding was received for this manuscript.

## Disclosure

We confirm that no individuals or third‐party services were involved in the research or manuscript preparation other than those listed as authors or acknowledged.

## Ethics Statement

This study was conducted in accordance with the ethical standards of the Declaration of Helsinki. As this was a retrospective review of anonymized clinical data conducted within a private clinic, formal ethics committee approval was not required. All data were handled confidentially and in compliance with applicable data protection regulations.

## Consent

Written informed consent for publication was obtained from the patient.

## Conflicts of Interest

The authors declare no conflicts of interest.

## Data Availability

The data that support the findings of this study are available upon request from the corresponding author. The data are not publicly available due to privacy or ethical restrictions.
